# Anxiety in Children with Selective Mutism: A Meta-analysis

**DOI:** 10.1007/s10578-019-00933-1

**Published:** 2019-10-24

**Authors:** Jim Driessen, Jan Dirk Blom, Peter Muris, Roger K. Blashfield, Marc L. Molendijk

**Affiliations:** 1 Faculty of Social and Behavioural Sciences, Leiden University, Wassenaarseweg 52, 2333 AK Leiden, The Netherlands; 2 Parnassia Psychiatric Institute, Kiwistraat 43, 2552 DH The Hague, The Netherlands; 3 Department of Psychiatry, University Medical Centre Groningen, University of Groningen, Hanzeplein 1, 9700 RB Groningen, The Netherlands; 4 Faculty of Psychology and Neuroscience, Maastricht University, Maastricht, The Netherlands; 5 Stellenbosch University, Stellenbosch, South Africa; 6 Auburn University, 226 Tach Hall, Auburn, AL 36849 USA; 7 Leiden University Medical Center, Albinusdreef 2, 2333 ZA Leiden, The Netherlands

**Keywords:** Selective mutism, Elective mutism, Anxiety disorder, Meta-analysis, DSM-5

## Abstract

This study evaluates the current conceptualization of selective mutism (SM) as an anxiety disorder in the DSM-5 using a meta-analytic approach. In the absence of any systematic assessment of anxiety in the field of SM, we pooled prevalence data of comorbid anxiety disorders in a random-effects meta-analysis. On the basis of 22 eligible studies (*N* = 837), we found that 80% of the children with SM were diagnosed with an additional anxiety disorder, notably social phobia (69%). However, considerable heterogeneity was present, which remained unexplained by a priori specified moderators. The finding that SM is often diagnosed in combination with anxiety disorders, indicates that these disorders are not discrete, separable categories. Moreover, this finding does not help to elucidate the relation between SM and anxiety as an etiological mechanism or symptomatic feature. Broadening our research strategies regarding the assessment of anxiety is paramount to clarify the role of anxiety in SM, and allow for proper classification.

**Electronic supplementary material:**

The online version of this article (10.1007/s10578-019-00933-1) contains supplementary material, which is available to authorized users.

## Introduction

Selective mutism (SM) is a psychiatric disorder that is usually—although not necessarily—diagnosed during childhood. According to the latest edition of the *Diagnostic and Statistical Manual of Mental Disorders* (DSM-5 [1]) it is characterized by a consistent failure to speak in specific social situations where there is an expectation for speaking (e.g., at school), while the production of speech appears to be normal in other situations (e.g., at home). Therefore, SM cannot be attributed to a disturbance of language development. Nevertheless, the lack of speech usually interferes with occupational or educational achievement, and—obviously—with social communication, thus making SM a debilitating disorder. The mean age of onset of SM is between 2.7 and 4.6 years [2–5], although the condition may go unnoticed until the child enters elementary school [6]. SM appears to be slightly more common in girls than in boys, with a reported sex ratio between 1:1.2 and 1:2 [7–9]. Most attempts to estimate the prevalence of SM have been conducted in school-based communities and yielded point prevalence rates ranging between 0.03 and 0.79% [4, 10–13]. SM is therefore considered to be a relatively rare psychiatric disorder [1].

The origin of the concept of SM has often been traced to Kussmaul (1822–1902), the German physician still known today for Kussmaul’s sign, Kussmaul breathing, and a host of non-eponymous terms with ongoing clinical relevance. His 1877 pioneering work on speech disturbances offered several clinical descriptions of “absence of speech without disturbance of speech”, a condition he associated primarily with cases of “hysteria and other neuroses” (p. 200) [14]. However, in the literature that followed, a subtle yet oft-repeated misconception has been that Kussmaul called this condition ‘aphasia voluntaria’ (i.e., ‘voluntary inability to speak’), a malapropism of the term ‘aphrasia voluntaria’ that he actually used, which has the more plausible meaning of a ‘voluntary absence of speech’ (p. 211) [14]. Apart from introducing this term, Kussmaul positioned *aphrasia voluntaria* among other speech disturbances, thus providing an extensive frame of reference for differential diagnosis. Still, as indicated by his lavishly referenced book, he was not the first and certainly not the last to describe this condition and come up with a name for it.

In 1934 another pioneer in the area of speech disorders, the Swiss child psychiatrist Tramer (1882–1963), suggested *elektiver Mutismus* as an appropriate term. As he expounded in his detailed case report of a seven-year-old boy, “Since the mutism [of this boy] was restricted to contacts with a (subconsciously) chosen group of people, I would like to propose the name elective mutism to designate it” [15]. In Tramer’s view, children with this diagnosis are not aphasic, but deliberately *choose* to remain silent [15, 16]. And yet, almost as an afterthought, he also mused that the boy described in his paper had made a “catatonia-like impression” during his phases of mutism [15].

Even though Tramer’s contribution only consisted of a case report, the term *elektiver Mutismus* was eventually adopted almost universally. It made its first entrance in the English literature as ‘elective mutism’ (EM) when Salfield [17] published a case report of another 7-year-old boy who never spoke at school. Salfield suggested that the problem might well have been related to a malfunctioning family system, a sociodynamic interpretation that was in vogue at that time [18–23], but had already been suggested as a possible mechanism by Tramer [15]. Perhaps the principal merit of Tramer’s paper was that it included such a scholarly overview of the mechanisms thought to be underlying the problem, ranging from hereditary factors and delayed phases of normal child development (‘being mummyish’) to psychological factors (e.g., shyness, hypersensitivity), social mechanisms (e.g., social anxiety, early childhood trauma, learned behavior in reaction to external pressure), physical conditions (e.g., surgery), and major psychopathology (e.g., oligophrenia, schizophrenia, catatonia). Even therapeutically, Tramer showed himself ahead of his time, preluding on many of the behavioral techniques that would later be advocated by psychotherapists for treating children with SM [16, 24–27].

Quantitative empirical research in the field of EM remained sparse in the following decades. Only eight studies had examined samples of 10 or more children with EM prior to 1991 [28]. Moreover, research on this topic had been using widely varying diagnostic criteria and adopted different explanatory models of the condition [29]. As noted by Tancer [9], this may have led to the grouping together of children with heterogeneous types of underlying pathology, and hence to (partly) conflicting descriptions of EM in the literature. There were even studies in which these children were labeled as passive-aggressive, stubborn, manipulative, oppositional, and/or controlling [18, 19, 22, 23, 28], although others described them the way Tramer [15] had done, i.e., as being overly shy, dependent, anxious, and/or hypersensitive [17, 25, 26, 30, 31].

EM was first included as a diagnostic category in the DSM-III, under the general heading of *Other Disorders of Infancy, Childhood, or Adolescence* [32]. In conformity with its overall ‘atheoretical’ approach, the DSM Task Force for DSM-IV decided to replace ‘Elective Mutism’ by ‘Selective Mutism’ (SM), thus expressing the notion that this type of mutism is found in specific contexts rather than being (necessarily) self-chosen [33]. Along with this terminological change, a growing number of researchers began to suggest that SM may be primarily related to anxiety [2, 34–38], and should therefore be relocated to the group of anxiety disorders. Black and Uhde [2] were among the first to provide support for this claim when they found that 97% of the children with SM also met the diagnostic criteria for either social phobia or avoidant personality disorder. A substantial number of empirical studies reporting that symptoms of anxiety are indeed often present in children with SM have since been published [7, 39–42]. Therefore, the plan to reclassify SM as an anxiety disorder was effectuated in the DSM-5 [1].

The relationship between SM and anxiety was reviewed by several authors, including Muris and Ollendick [41], who concluded that both conditions tend to overlap in terms of symptomatology, etiology, and treatment approaches. However, the precise nature of this relationship is still insufficiently clear. A latent-profile analysis by Cohan et al. [7] revealed three subtypes of SM, each indicating a clinically significant presence of social anxiety. Several studies even reported significantly higher observer ratings of anxiety for children with SM in comparison to children with social phobia [43–45], although this finding has not been documented in all studies [46, 47]. Moreover, children diagnosed with SM do not report higher levels of anxiety than children with social phobia on self-report measures [43, 44, 47, 48], nor do they display higher levels of anxiety when measured with the aid of psychophysiological assessment instruments [45]. Perhaps these contradictory findings are caused by an observer bias in which the observers seek to attribute mutism to (social) anxiety [43, 44]. Even though the overlap between SM and social phobia appears to be high, surprisingly low rates of comorbid social phobia have also been reported [49, 50], although part of the children in these studies had been diagnosed with other comorbid anxiety disorders. As a matter of fact, comorbid diagnoses of anxiety disorder other than social phobia are commonly reported in children with SM, including specific phobia [46, 50] and separation anxiety disorder [8, 51].

Remarkably however, anxiety—as a symptom—does not appear in the DSM’s diagnostic criteria for SM (Table 1), despite its reclassification as an anxiety disorder. As a consequence, perhaps, its presence has not always been assessed systematically in empirical studies of individuals diagnosed with SM. Instead, researchers have often sought to corroborate the link between SM and anxiety by focusing on the presence of comorbid anxiety disorders. This is not as plausible as it may sound, since an additional comorbid diagnosis of social anxiety disorder may indeed be suggestive of a common mechanism underlying the two disorders, whereas, for example, a specific phobia for spiders does not necessarily say something about one’s reluctance to speak in specific social situations. Nonetheless, since direct assessments of levels of anxiety in SM are extremely rare, the body of literature on comorbid anxiety disorders in SM may currently be the best proxy for establishing a link between SM and anxiety. To actually assess the strength of this link, the present study makes use of a meta-analysis. Its main purpose is to provide an overall estimation of the prevalence rate of comorbid anxiety disorders in children with SM.

**Table 1:** Current diagnostic criteria for selective mutism as described in the DSM-5 [1]

Diagnostic criteria	312.23 (F94.0)
A.	Consistent failure to speak in specific social situations in which there is an expectation for speaking (e.g., at school) despite speaking in other situations
B.	The disturbance interferes with educational or occupational achievement or with social communication
C.	The duration of the disturbance is at least 1 month (not limited to the first month of school)
D.	The failure to speak is not attributable to a lack of knowledge of, or comfort with, the spoken language required in the social situation
E.	The disturbance is not better explained by a communication disorder (e.g., childhood-onset fluency disorder) and does not occur exclusively during the course of autism spectrum disorder, schizophrenia, or another psychotic disorder

## Method

### Search Strategy

A systematic search was performed to identify all empirical studies pertaining to the topic of SM. Historically, the terms “elective mutism”—and later—“selective mutism” have been used almost exclusively to refer to this psychiatric disorder, and were correspondingly adopted by diagnostic classification systems such as the ICD and the DSM. We therefore argued that the inclusion of these two terms alone in our search query should provide a scope broad enough to capture all potential publications in this field of research. Accordingly, the digital databases *Web of Science*, *PubMed*, *PsycINFO*, *Embase*, and *Picarta* were searched from their inception through March 2019 using the following search query: ‘[*elective* OR *selective* AND *mutism*]’. Our search strategy was supplemented by backward searches in which the reference lists of the retrieved papers were screened for publications that were not detected by the initial search.

### Study Selection Procedure

All obtained references were stored and managed in EndNote X9 software. Duplicate records were identified and carefully removed from the initial search result. The first author (JD) explored all unique references by screening the titles and applying filters on record properties, such as language, keywords, and type of resource. Publications not written in English were excluded, along with resources that were clearly unsuitable for inclusion in the meta-analysis (e.g., book reviews or commentaries). Non-peer-reviewed resources (e.g., conference abstracts or dissertations) were also excluded, in order to maintain the scientific value of the results derived from our meta-analysis. All remaining records were subjected to abstract-review. A reference was considered potentially relevant when there was an indication of a sample of individuals diagnosed with SM being described in the paper. Potentially relevant papers that were selected were subsequently full-text reviewed to determine if they met the following criteria for eligibility: (i) the study reported on a sample of individuals diagnosed with SM; (ii) the researchers reported the necessary data for extraction—particularly those related to comorbid anxiety—or provided these after a request hereto via email; (iii) the study reported original data on individuals diagnosed with SM. Full-text reviews were carried out by two authors (JD and MLM) independently, with disagreements being resolved through discussion until consensus was reached.

### Data Extraction

The following data was extracted from eligible studies: (i) year of publication; (ii) country and setting of recruitment; (iii) number of participants; (iv) distribution of sex and age among the sample; (v) diagnostic criteria and instruments used to diagnose SM; (vi) prevalence rates of comorbid anxiety disorders. Data extraction was initially performed by the first author (JD). In order to enhance the quality of the collected data, a second researcher (MLM) extracted data from a random subset of 25% of the eligible studies and blindly checked both extractions for accuracy. Possible discrepancies in extracted data were resolved by consensus between the two authors.

### Quality Assessment

All eligible studies were assessed for their methodological quality using the Newcastle-Ottawa Scale (NOS) [52]. Since we performed a meta-analysis of prevalence, research methodology with respect to sample selection and baseline assessment were deemed most relevant for quality evaluation. We therefore adapted the NOS to focus on these aspects only (see Online Supplement for our modified version). The maximum quality assessment score of 12 points corresponded to excellent methodological quality, whereas a score of 0 indicated poor methodological quality. Assessment of quality was performed by two authors independently (JD and MLM). Agreement regarding this assessment proved to be good among the two independent raters (Cohen’s Kappa [κ] = 0.67; Standard Error [SE] = 0.059).

### Calculation of Comorbid Anxiety

Studies had to report on the presence of comorbid anxiety disorders as established with the aid of validated instruments in order to be included in the meta-analysis, such as (semi-) structured interviews. We did not discriminate with regard to severity, type or number of anxiety disorders that were diagnosed in children with SM. Whenever an indicator of anxiety among the sample was reported, then this proportion was directly implemented. However, most studies reported only frequencies of additionally diagnosed comorbid anxiety disorders. Studies were excluded from the meta-analysis when we were unable to ascertain the true proportion of anxiety and/or the presence of comorbid anxiety disorders, either through calculation or through a request via email to the original authors. Exceptions were made for studies in which a diagnosis of one type of anxiety disorder was highly overrepresented in the study sample, while the sum of diagnosed anxiety disorders did not exceed the sample size. Under these conditions, we argued that using the sum of additionally diagnosed anxiety disorders was sufficient to determine the total proportion of comorbid anxiety disorders in the study sample.

### Statistical Analysis

All statistical calculations were performed using *Metafor*, a meta-analysis package for *R Statistics Software* [53, 54]. We employed a random-effects meta-analysis to estimate the aggregate prevalence rate of comorbid anxiety disorders in children with SM. A restricted maximum-likelihood estimation was used in the model, since this estimator is efficient and approximately unbiased [55]. Variance was stabilized using a double-arcsine transformation [56, 57], which is the preferred method when prevalence rates are used as a variable in meta-analyses [58]. The results were back-transformed for interpretational purposes.

Possible heterogeneity was assessed for statistical significance using the *Q*-statistic and evaluated using *I*^2^. High values of *I*^2^ indicate increased between-study heterogeneity [59], in which case a meta-regression analysis would be performed to explore for possible moderating effects. We a priori specified potential moderators, which included *age*, *gender*, *year of publication*, and *methodological quality*. We controlled for Type-I-error rate using the Knapp and Hartung adjustment [60], which shows higher power rates and better correction to the nominal significance level than the standard method [61, 62].

Publication bias refers to the observation that studies yielding significant results are more likely to be published than studies yielding null results [63]. Assessment for publication bias was deemed not of interest in the context of our meta-analysis, since prevalence rates were used as a variable rather than outcome measures. That is to say, we assumed that publication in the field of SM would not depend on the observed prevalence of comorbid anxiety disorders, as it is unlikely that studies manipulated variables in order to achieve a certain prevalence rate of comorbid anxiety disorders in their sample. Therefore, null findings are likely nonexistent when prevalence rates are used as a variable, which makes it redundant to assess for publication bias.

## Results

### Search Results

**Fig. 1:**
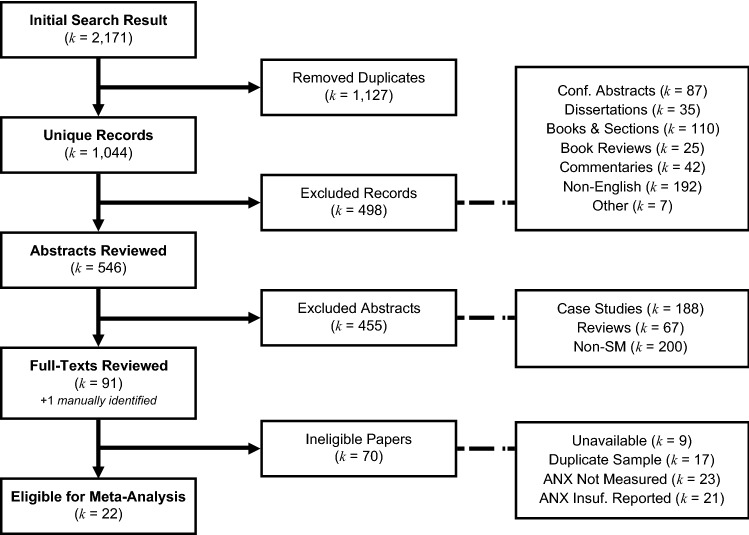
Flowchart of the study selection procedure in (k) number of publications

A flowchart of the study selection procedure is depicted in Fig. 1. Our search strategy identified 1044 (*k*) unique records. After a first screening of titles, keywords, and resource types, we filtered out 498 of these records, either because they were not written in English (*k* = 192) or because they indicated a type of resource that was considered unsuitable for inclusion in the meta-analysis (*k* = 306). Abstracts of the remaining 546 references were reviewed in order to identify studies examining samples of children diagnosed with SM. A total number of 200 abstracts did not mention SM as a main topic of interest, and were therefore excluded, although they sometimes did refer to SM in one way or another. Most of these excluded articles focused on anxiety disorders in general (22%) or on social phobia (9.5%). SM was also mentioned in the context of schizophrenia (8.5%), autism (6.5%), linguistic-related dysfunction (7%), and a wide range of neurological diseases (15.5%). The 346 references that remained after these exclusions comprise the entire English peer-reviewed literature on the topic of SM as revealed by our search strategy. The majority of these reports consisted of case studies (*k* = 188) or reviews (*k* = 67), which were unsuitable for meta-analysis, and therefore also excluded. Further screening via abstract review identified 91 papers reporting on samples of individuals diagnosed with SM that were retained for full-text review, along with one paper that was detected with the aid of backward searches [8]. Two authors (JD and MLM) independently carried out the full-text reviews for eligibility. Only minor disagreements occurred, which were easily resolved after consensus was reached. Finally, a total of 22 studies fulfilled the inclusion criteria for eligibility to participate in the meta-analysis.

### Study Characteristics

All necessary data for meta-analysis was extracted from the 22 eligible studies by the first author (JD) and blindly checked by a second author (MLM). Following this procedure, no discrepancies were found. Collectively, the 22 studies comprised a combined sample size of *N* = 837 children diagnosed with SM. The majority of the total sample was female (*n* = 499), and the average age was 8.2 years (*SD* = 3.14; *k* = 20; two papers did not report on this). Table 2 provides an overview of the demographic and clinical characteristics of the children included in these 22 studies. With respect to sample selection, most children had been recruited from school-based communities and/or clinics specialized in child psychiatry. A diagnosis of SM was generally ascertained with the aid of a (semi-)structured interview with the parents of the participating child. All studies relied on DSM-IV diagnostic criteria, except for one study that used ICD-10 criteria [64]. More detailed information regarding the recruitment and assessment strategies is provided in the online supplement (Table S1). Eligible studies were evaluated for their methodological quality using an adapted version of the NOS. The average quality rating of the included studies was 58.2% of the maximum score (*M* = 6.9; *SD* = 2.21). Items related to sampling methodology had an average rating of 52% (*M* = 3.1; *SD* = 1.34), whereas items related to baseline assessment had an average quality rating of 64% of the maximum score (*M* = 3.8; *SD* = 1.58).

**Table 2:** An overview of the eligible studies, including sample characteristics and reported prevalence rates of anxiety disorders

Study	N	Gender		Age	Anxiety disorders (%)						*QR*
		M	F	*M (SD)*	ANX	SOP	SAD	SPH	GAD	OCD	
Alyanak et al. [65]	26	11	15	8.11 (2.1)	73.1	61.5	23.1				0.58
Andersson and Thomsen [64]	37	20	17	9.43 (3.8)	59.5^a^	45.9	8.1		0.0	8.1	0.50
Arie et al. [66]	18	8	10	8.89 (1.9)	50.0^a^	44.4	5.6				0.46
Bar-Haim et al. [67]	16	5	11	8.21 (3.5)	87.5^a^	62.5					0.29
Black and Uhde [2]	30	9	21	8.40 (2.0)	96.7	96.7	16.7		10.0	3.3	0.75
Carbone et al. [39]	37	18	19	8.20 (3.4)	75.6	18.2	13.6	29.5	2.3		0.38
Chavira et al. [51]	70	26	44	6.37 (2.5)	100.0	100	40.0		11.4	8.6	0.63
Cholemkery et al. [46]	43	26	17	11.09 (3.9)	44.2		4.7	32.6			0.88
Dummit et al. [68]	50	14	36	8.20 (2.7)	100.0	100	26.0		14.0		0.88
Edison et al. [49]	21	13	8		33.3	14.3	14.3	14.3			0.33
Gensthaler et al. [69]	95	47	48	9.70 (4.5)	93.7	93.7	20.0	21.1	5.3		0.58
Henkin et al. [70]	10	3	7	9.35 (2.6)	70.0^a^	60.0	10.0				0.42
Kristensen et al. [3]	54	22	32	9.00 (3.4)	74.1	66.7	31.5	13.0	13.0	9.3	0.88
Lang et al. [71]	24	12	12	6.40 (3.1)	100.0	100.0	41.7	45.8	4.1		0.58
Levin-Decanini et al. [72]	48	13	35	6.53 (2.6)	58.3				6.3		0.71
Manassis et al. [40]	44	12	32	7.87 (1.6)	63.6^a^	61.4		2.3			0.75
Mulligan et al. [8]	142	52	90		29.6						0.50
Nowakowski et al. [50]	14	6	8	6.36 (0.9)	50.0	0.0	21.1	21.1			0.29
Oerbeck et al. [73]	24	8	16	6.50 (2.0)	100.0	100.0	29.2	25.0	8.3	8.3	0.75
Vecchio and Kearney [42]	15	7	8	6.58 (1.9)	100.0	100.0	40.0	20.0	6.7	0.0	0.67
Vecchio and Kearney [74]	9	2	7	6.60 (1.9)	100.0	100.0	22.2	22.2	11.1		0.54
Young et al. [45]	10	4	6	7.00 (1.8)	80.0	80.0	0.0	0.0	0.0		0.46

*ANX* anxiety disorders, *SOP* social phobia, *SAD* separation anxiety disorder, *SPH* specific phobia, *GAD* generalized anxiety disorder, *OCD* obsessive-compulsive disorder, *QR* quality rating expressed as a proportion of the maximum score

^a^The total proportion of comorbid anxiety disorders is determined by the sum of single anxiety disorders

### Meta-analysis

**Fig. 2:**
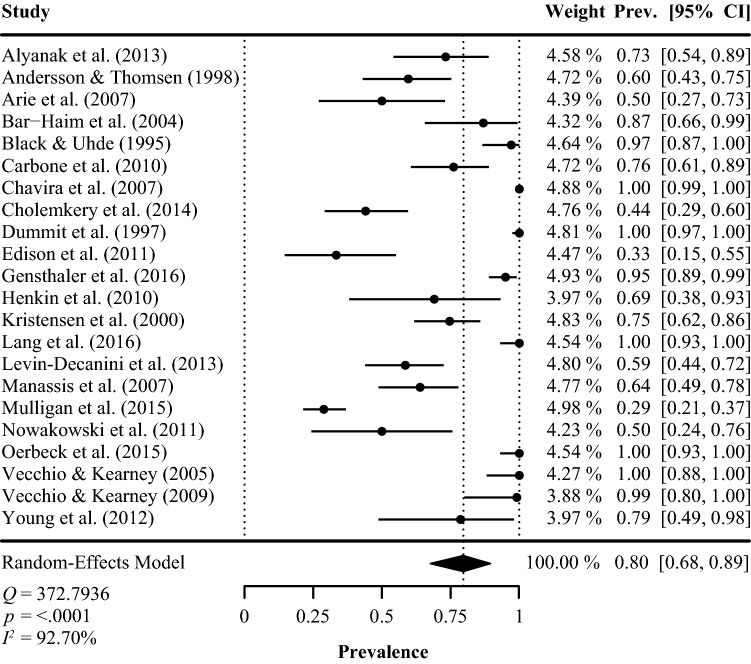
Forest plot for random-effects meta-analysis of the prevalence of comorbid anxiety disorders in children diagnosed with SM

The results of the primary meta-analysis are presented in Table 3 and depicted in Fig. 2. The harmonic mean proportion of children diagnosed with at least one additional (comorbid) anxiety disorder was 0.80 (95% CI = 0.68, 0.89). Considerable heterogeneity in prevalence figures was observed between the studies (*I*^2^ = 92.70, *p* <0.001). Prevalence rates for specific types of anxiety disorder among children with SM indicate that social phobia (i.e., social anxiety disorder) was the most commonly reported anxiety disorder across all studies (0.75, 95% CI = 0.56, 0.90). However, this outcome may be prone to bias due to missing data, as three studies did not specify the proportion of children with an additional diagnosis of social phobia. We therefore performed an imputed case analysis in which these missing values were filled in under the assumption that 75% of the children with comorbid anxiety disorder in these three studies had actually been diagnosed with social phobia. The imputed case analysis resulted in an aggregate prevalence rate of 0.69 (95% CI = 0.52, 0.84) of children with SM who were also diagnosed with social phobia.

**Table 3:** Results of the meta-analysis for the prevalence of (comorbid) anxiety disorders in children diagnosed with SM

Meta-analysis	*k*	Random-effects		Heterogeneity		
		Prevalence	CI (95%)	*Q*	*p*	*I*^2^ (%)
ANX	22	0.80	[0.68, 0.89]	372.79	< 0.001	92.70
SOP	19	0.75^a^	[0.56, 0.90]	348.98	< 0.001	95.29
SAD	18	0.18	[0.12, 0.24]	49.26	< 0.001	64.77
SPH	12	0.19	[0.12, 0.28]	34.65	< 0.001	69.24
GAD	13	0.06	[0.04, 0.09]	15.58	0.21	28.56
OCD	6	0.06	[0.03, 0.10]	2.71	0.74	0.00

*ANX* anxiety disorders, *SOP* social phobia, *SAD* separation anxiety disorder, *SPH* specific phobia, *GAD* generalized anxiety disorder, *OCD* obsessive-compulsive disorder

^a^0.69 after imputed case analysis CI (95% CI = 0.52, 0.84)

### Moderator Analysis

The heterogeneity in the prevalence rates of comorbid anxiety disorders among studies in the meta-analysis was considered very high [59]. In an attempt to explore the sources of this between-study heterogeneity, a moderator analysis was conducted by means of a mixed-effects-model meta-regression. A priori potential moderators included the mean age of the sample, the proportion of female participants, the year of publication, and the methodological quality of the study at hand. However, results of the meta-regression indicated that none of the moderating factors had a significant effect on the presence of comorbid anxiety disorders.

## Discussion

To our knowledge, the association between SM and anxiety (as a symptom) has never been investigated through a meta-analytic approach, despite its reclassification as an anxiety disorder in the DSM-5. The present study sought to examine the supposed link between SM and anxiety by means of a meta-analysis. Since levels of anxiety in children diagnosed with SM have not always been assessed systematically throughout the literature, data on comorbid anxiety disorders in SM was judged to be the best substitute for examining this relationship. Hence, the main purpose of the present study was to provide an overall estimation of the prevalence rate of comorbid anxiety disorders in children with SM on the basis of meta-analytic data.

A total of 22 studies met the eligibility criteria for inclusion in the meta-analysis, comprising data on 837 children diagnosed with SM. The results indicated that 80% of these children were additionally diagnosed with at least one comorbid anxiety disorder. Social phobia (i.e. social anxiety disorder) was found present in 69% of the children with a diagnosis of SM, making it the most commonly diagnosed comorbid anxiety disorder. This was followed by specific phobia (19%), separation anxiety disorder (18%), generalized anxiety disorder (6%), and obsessive-compulsive disorder (6%). By and large, these figures are in line with the observation made in the DSM-5 that, “In clinical settings, children with selective mutism are almost always given an additional diagnosis of another anxiety disorder—most commonly, social anxiety disorder” (p. 196) [1]. However, they cannot fully support the notion that anxiety is always present in SM, as is currently implied by its classification as an anxiety disorder.

### Etiological Heterogeneity

While diagnoses of comorbid anxiety disorder may account for the presence of anxiety in 80% of the children diagnosed with SM, it remains unclear how anxiety is manifested in the remaining 20% of the children that lacked such an additional diagnosis. The first and rather obvious reason for this is that anxiety does not feature in the DSM-5’s list of diagnostic criteria for SM, and that in clinical practice it is hardly ever assessed in a direct way. Moreover, the presence of a comorbid anxiety disorder does not automatically imply that SM originates from the same source (i.e., anxiety)—that is to say, not any more than the presence of a comorbid depressive disorder would imply that SM would be caused by depression. As noted by Tramer as early as 1934, numerous factors are to be taken into consideration if we want to unravel the (probably heterogeneous) etiology of SM [15]. Preferably, one would like to investigate the presence or absence of these factors in populations with SM in a direct way. Regarding anxiety levels, this should ideally be done with the aid of validated questionnaires and psychophysiological measurements of heart rate, blood pressure, and skin conductance, or some other method to assess physiological arousal. Since in clinical practice such assessments are hardly feasible because of the very problem at hand (i.e., mutism and uncooperativeness), the assessment of comorbid anxiety disorders has been embraced as a litmus test for the presence of anxiety. However, our analysis indicates that such comorbid anxiety disorders are undiagnosable in 20% of all children with SM. Moreover, among the remaining 80% there is a group of no less than 19% with a fear for flying, heights, animals, receiving an injection, seeing blood, and so on (i.e., specific phobias). One may ask oneself whether the anxiety underlying these specific phobias is really sufficient to explain a lack of speech in front of specific individuals. In sum, by focusing exclusively on comorbid anxiety disorders, the DSM would appear to restrict its approach to ‘looking where the light shines’. This brings us back to Tramer’s observation that the etiology of SM is likely to be diverse. When Tancer [9] reviewed the literature to facilitate modifications of the diagnostic criteria of SM in the DSM-IV, she argued that the criteria had been defined very broadly. Like Tramer [15] before her, she expressed the suspicion that this was likely to result in the inclusion of children with diverse underlying pathology. Consequently, she emphasized that systematic research was needed before more specific criteria could be designed. Now, more than 25 years later, the number of studies investigating samples of children with SM has grown substantially. And yet Tancer’s concern remains topical as the DSM criteria of SM have not been changed, despite its new classification as an anxiety disorder.

### Consequences for Classification

Our lack of insight into the full spectrum of etiological mechanisms underlying SM has several consequences for the classification of SM in the DSM and other taxonomies. On the basis of our meta-analysis and subsequent discussion, we conclude that we are currently not in a position to establish with sufficient accuracy whether anxiety plays a key role in all cases of SM, or even in a majority of them. From that vantage point, it is as yet uncertain how SM should be classified (i.e., as a member of the higher-level group of anxiety disorders, or perhaps as a member of the groups of communication disorders, oppositional defiant disorders or neurodevelopmental disorders, to mention some of the various possibilities). The so-called ‘atheoretical’ approach of the DSM has been much debated, but in conformity with this vantage point it might well have been more appropriate to stick to the classification of SM as a disorder of infancy, childhood, or adolescence (i.e., what is currently called a neurodevelopmental disorder in the DSM-5). After all, by relocating SM to the group of anxiety disorders, the implicit theoretical assumption appears to have been made that anxiety constitutes the disorder’s symptomatological and/or etiological cornerstone. Since the DSM-5 does acknowledge the issue of etiological heterogeneity by pointing out the influence of temperamental, environmental, genetic, and physiological factors, albeit under the heading of Risk and Prognostic Factors, it is all the more remarkable that SM ended up under the heading of Anxiety Disorders.

That said, the considerable overlap of SM with social phobia may also suggest that these two disorders, as defined in the DSM-5, are not discrete, separable categories. Psychiatric classification is different from biological classification, but an analogy may nonetheless be illuminating. For example, when defining the housecat as a species, biologists do not have the habit of listing all its characteristic features (i.e., having fur, walking on four legs, purring when petted, being good at ignoring people yet being friendly to others, etc.). Instead, they focus on the features that separate housecats from the other species within the overarching genus *Felis*. They do not list the features that all cats (including lions, tigers, etc.) have, or all mammals have, etc. In the DSM-5, “Anxiety Disorders” is a family-level category somewhat like “mammal”. In accordance with the principles of biological classification, if SM fits under the category of anxiety disorders, then persons with SM should also meet the more general definition of an anxiety disorder. As a consequence, the definitional task here would not be to simply describe SM, but, instead, to define whatever characteristics separate SM from the other anxiety disorders, especially social phobia [75]. The present study indicates that the current DSM definition of SM does not meet this goal. Sticking with the example of biological classification, the question whether SM is an anxiety disorder, should then become whether most children with SM meet the definition of any of the higher-level groups of disorders.

### SM in Other Age Groups

By focusing almost exclusively on children, the DSM-5 also precludes the possibility of a proper comparison of SM across age groups. Clinical practice teaches us that SM is also found in the context of schizophrenia spectrum disorders, catatonia [76], autism [77], and numerous other disorders in adulthood, and even outside the domain of psychopathology, where it may serve as a powerful tool to vent one’s misapprehension of—or hostility towards—another person; or, alternatively, be an expression of extreme shyness or insecurity. Although mutism has been studied in adult populations, notably in the context of autism and catatonia, there is a dire need to focus such studies on the presence of SM if we wish to say more about a possible continuum with the population targeted by the diagnostic category of SM in the DSM-5. As pointed out by Kussmaul as early as 1877, the essence of SM appears to be that it is a speech disorder. The diagnostic criteria of SM in the DSM-5 seem to be most in line with this viewpoint. As a consequence, it may perhaps be necessary to revise its current classification as an anxiety disorder—at least until empirical research provides us with data to back up an alternative choice, and the classificatory issues regarding the defining characteristics of higher-level groups of disorders and the distinguishing features of lower-level, individual disorders has been solved.

### Limitations

Our results should be viewed in light of several limitations. The initial search strategy identified a large number of records that had to be filtered out before abstracts were reviewed, which partly included non-peer reviewed material, such as conference abstracts and dissertations. Although we argue that the exclusion of these resources improved the scientific value of our meta-analysis, there is a possibility that important data on groups of children diagnosed with SM went undetected due to this procedure. The same holds for publications not written in English, which comprised nearly one-fifth of the records identified by our initial search strategy.

Another limitation is the observed heterogeneity of prevalence figures, as this impedes the drawing of conclusions from the meta-analysis. Heterogeneity between studies is likely to arise from sampling errors and/or differences in research methodology across studies [59]. In case of the current study, these shortcomings may have been amplified by the broad scope of our search strategy. That is to say, our search query was defined rather broadly in order to capture all publications pertaining to the topic of SM, while the inclusion criteria for meta-analysis made no requirements regarding research design or research objective, since prevalence rates were used as a variable, rather than outcome variables. Consequently, different types of study were deemed eligible for inclusion in the meta-analysis, which likely resulted in varying diagnostic assessment strategies among the studies. We did not exclude papers on the basis of methodological quality due to the small size of the literature on the topic of SM. However, evaluation of methodological quality was rated only moderate on average and, indeed, revealed varying results across the studies. Although this may simply reflect the current state of research in the field of SM, sampling errors and differences in research methodology were likely present and may thus have contributed to the observed heterogeneity.

To deal with this, we explored possible moderating effects through meta-regression analysis. Extracted data enabled us to include *age*, *proportion of female subjects*, and *year of publication* as potential predictors in the equation. Additionally, we included *methodological quality* as a moderator on the basis of the results of the quality assessments. However, the results of our mixed-effects meta-regression analysis revealed that none of these moderators could explain a significant part of the heterogeneity that was observed in the meta-analysis.

### Recommendations for Clinical Practice and Research

Several recommendations can be made following the results of our study. First of all, children with SM should be assessed in greater detail with regard to the anxiety-related symptoms they may experience, including concrete events that are being feared, and the content of associated cognitions. Such an extensive assessment is preferably to be supported by objective measures (e.g., psychophysiological measurements), since subjective assessments made by parents, teachers or clinicians are not sufficient in this respect, especially when children with SM remain silent, and may not even be in a position to confirm or deny whether any of the conclusions tally with their own experiences [45]. Secondly, we need to broaden the scope of these assessments so as to include other factors that might play a role in the etiology of SM, including the temperamental, environmental, genetic, and physiological factors mentioned in the DSM-5. Thirdly, future research could enlarge the target population by also including adolescents, adults, and elderly people, in whom SM has remained largely unexplored. In the fourth place, we advocate the development of standardized questionnaires and psychophysiological measurements so as to promote the homogeneity between studies and increase overall reliability, particularly when clinical anxiety in children diagnosed with SM is being examined. Together, these steps should help us to elucidate the etiological factors underlying SM. Meanwhile, for as long as these remain uncertain, we advocate a revision of the current classification of SM to help us prevent making any premature associations with anxiety or certain age groups, and also to help us to keep an open eye for a possible differentiation of the condition into several subtypes.

## Summary

Our meta-analysis indicates that SM has a relatively high association with comorbid anxiety disorders. Nonetheless, it also indicates that the presence of these comorbid disorders fails to validate the presence of an additional anxiety disorder in all cases of SM, and that, moreover, the one-sided focus on comorbid anxiety disorders obscures our view of the numerous other etiological factors that may be at play. As a corollary, we advocate a substantial broadening of our research strategies, a standardization of the assessment tools to be used, and the inclusion of other age groups than children alone, to further our understanding of SM. In the meantime, to prevent ourselves from jumping to conclusions, we advocate a revision of the current classification of SM in psychiatric classifications such as the DSM, and maintain focus on its core characteristic—that is failure of speech—at least until empirical research has caught up with our ideas about the origin of this curious and debilitating phenomenon, and several overarching classificatory issues have been solved.

## Electronic supplementary material

Below is the link to the electronic supplementary material.

Supplementary material 1 (PDF 410 kb)
