# Supplementary material for: Anxiety in Children with Selective Mutism: A Meta-analysis
Source: Child Psychiatry Hum Dev. 2019 Oct 24;51(2):330–41. doi: 10.1007/s10578-019-00933-1 (PMC7067754; doi:10.1007/s10578-019-00933-1)
Supplement: Supplementary file 1 — Supplementary material 1 (PDF 410 kb) [file 10578_2019_933_MOESM1_ESM.pdf]

Supplementary Material

Anxiety in Children with Selective Mutism: A Systematic Review and Meta-Analysis

Child Psychiatry & Human Development

Driessen, Blom, Muris, Blashfield & Molendijk

Corresponding authors

J. Driessen [1]
Email: *f.j.driessen@umail.leidenuniv.nl*

M. Molendijk [1,2]
Email: *m.l.molendijk@umail.leidenuniv.nl*
Phone: *+31 71 527 667*

[1.] Faculty of Social and Behavioural Sciences, Leiden University, Wassenaarseweg 52, 2333 AK Leiden, the Netherlands

[2.] Leiden University Medical Center, Albinusdreef 2, 2333 ZA Leiden, the Netherlands

Table S1. Characteristics of sample selection and diagnostic assessment for included studies.

			Assessment	
Study	Country	Sample Recruitment	SM	Anxiety
Alyanak et al. (2013)	Turkey	Admissions to the Department of Child and Adolescent Psychiatry at the Istanbul Faculty of Medicine between 2006 and 2010.	Series of non-standardized clinical interviews conducted by the senior author.	Series of non-standardized clinical interviews conducted by the senior author.
Andersson & Thomsen (1998)	Denmark	Referrals for treatment as in- or out-patients at the Psychiatric Hospital for Children and Adolescents in Aarhus, Denmark.	Blind retrospective screening for symptoms of elective mutism following ICD-10 criteria, done by two authors.	Blind retrospective screening for symptoms of anxiety following ICD-10 criteria, done by both authors.
Arie et al. (2007)	Israel	Respondents to an article on SM published in a nationally distributed magazine and to a national television segment.	Semi-structured interview (unspecified) with parents and home-made video/audio tapes evaluation.	Structured interview K-SADS-PL conducted with the parents.
Bar-Haim et al. (2004)	Israel	Respondents to an national television segment on SM.	Semi-structured interview (unspecified) with parents and home-made video/audio tapes evaluation.	Structured interview K-SADS-PL conducted with the parents.
Black & Uhde (1995)	United States	Mailing announcements to elementary school counselors in Maryland, Virginia and District of Columbia.	Parents' responses to the PARIS structured interview (adapted from the DISC).	Parents' responses to the PARIS structured interview (adapted from the DISC).
Carbone et al. (2010)	Canada	Children with SM consisted of referrals from children's mental health agencies in Southern Ontario.	Parent or teacher reports on the SSQ, indicating at least two situations of mutism.	Parents report on the computerized version of the DISC.
Chavira et al. (2007)	United States	Nationwide sample recruited from the SM Group-Child Anxiety Network and from parent oriented-conferences.	SM module of the ADIS-IV conducted with parents together with scores on the SMQ.	ADIS-IV conducted with parents.
Cholemkery et al. (2014)	Germany	Admissions to the Department of Child and Adolescent Psychiatry, Psychosomatics and Psychotherapy in Frankfurt am Main between 2011-2012.	Parents' responses to the Kinder-DIPS diagnostic screening interview.	Parents' responses to the Kinder-DIPS diagnostic screening interview.
Dummit et al. (1997)	United States	Subjects were recruited through advertising and referral from local schools and the Selective Mutism Foundation.	Parents' responses to the PARIS structured interview (adapted from the DISC).	Parents' responses to the PARIS structured interview (adapted from the DISC), and the L-SAS.
Edison et al. (2011)	Canada	Children were referred to a participating children's mental health referral site or recruited from a database of the McMaster University.	Parents or teachers reporting at least two situations of mutism on either the SSQ or the BCFPI.	Parents report on the computerized version of the DISC.

Table S1. Continued.

Study	Country	Sample Recruitment	Assessment	
			SM	Anxiety
Gensthaler et al. (2016)	Germany	Participants were part of another study on the psychopathology and diagnosis of SM conducted at universities in Germany.	Structured diagnostic interview (Kinder-DIPS) conducted with the mother of the child.	Structured diagnostic interview (Kinder-DIPS) conducted with the mother of the child.
Henkin et al. (2010)	Israel	Children were recruited through advertisement and through referrals from affiliated clinics.	Semi-structured interview (unspecified) with parents, evaluation of home-made video tapes and responses to the SMQ.	Structured interview (K-SADS-PL) conducted with the parents, The SCARED and SPAI-C questionnaires were utilized additionally.
Kristensen et al. (2000)	Norway	Mailing announcements to all 63 outpatient clinics for child and adolescent psychiatry and all 278 psychology services in Norway.	Telephone interview with the referring therapist ascertained a diagnosis of SM. Parents were subjected to an interview that followed the recommendations of Dow et al. (1995).	Diagnostic questions derived from the CAS parent version.
Lang et al. (2016)	Israel	Children that were being treated for SM in a clinic specialized in anxiety disorders and SM.	Parents were interviewed using the ADIS-IV. The SMQ was utilized to measure SM symptom severity.	Parents were interviewed using the ADIS-IV.
Levin-Decanini et al. (2013)	United States	Participants were seeking services at an urban mental health clinic that treats anxiety disorders in	Semi-structured interview ADIS-IV, parent and child version.	Semi-structured interview ADIS-IV, parent and child version.
Manassis et al. (2007)	Canada	Recruitment through clinics for anxiety disorders in three urban areas.	ADIS-IV interview with parents. Information from the SMQ was obtained from parents and teachers.	ADIS-IV interview with parents. Teachers' and parents' responses to SASC-R and MASC rating scales.
Mulligan et al. (2015)	United States	Subjects were derived from an archival database of the SM Anxiety Research and Treatment Center located in Southeastern Pennsylvania.	SM-CDQ parent report instrument for identification of SM symptoms and diagnosis.	The anxiety subscale of the SM-CDQ parent report instrument.
Nowakowski et al. (2011)	Canada	Recruited from local mental health agencies and from the McMaster University Child Database in Ontario.	Parent or teacher reports on the SSQ, indicating at least two situations of mutism.	Primary caregiver report on the internalizing section of the C-DISC.
Oerbeck et al. (2015)	Norway	Recruited from Child and Adolescent Mental Health Clinics and School Psychology Services in Southern Norway.	SM module of the ADIS-IV semi-structured interview.	Revised version of the K-SADS-PL.

Table S1. Continued

Study	Country	Sample Recruitment	Assessment	
			SM	Anxiety
Vecchio & Kearney (2005)	United States	Voluntary participants referred from elementary schools, preschools and Child and Family Services in southern Nevada.	SM Module of semi-structured interview ADIS-IV parent and child versions.	Semi-structured interview ADIS-IV parent and child versions.
Vecchio & Kearney (2009)	United States	Subjects were recruited from public and schools, preschools and from a general press release to the community.	Formal assessment based on a battery of tests including CBCL and TRF.	Semi-structured interview ADIS-IV, parent and child versions.
Young et al. (2012)	United States	Recruited from flyers sent to schools and public libraries describing SM and offering free assessment.	Semi-structured interview ADIS-IV with parents.	Completion SPAI-C by parents and children, and semi-structured interview ADIS-IV with parents.

Note. ICD-10 = international statistical classification of diseases and related health problems; K-SADS-PL = schedule for affective disorders an schizophrenia for school-age children, present and lifetime version; PARIS = parent as respondent informant schedule; DISC = diagnostic interview schedule for children; SSQ = speech situations questionnaire; ADIS-IV = anxiety disorders interview schedule for dsm-iv; SMQ = selective mutism questionnaire; Kinder-DIPS = diagnostic interview for psychiatric disorders in children (german); L-SAS = liebowitz social anxiety scale; BCFPI = brief child and family phone interview; SCARED = screen for child anxiety-related emotional disorders; SPAI-C = social phobia and anxiety inventory for children; CAS = child assessment schedule; SAS-R = social anxiety scale for children revised; MASC = multidimensional anxiety scale for children; SM-CDQ = selective mutism comprehensive diagnostic questionnaire; C-DISC = computerized diagnostic interview schedule for children; CBCL = child behavior checklist; TRF = teacher report form.

NEWCASTLE-OTTAWA QUALITY ASSESSMENT SCALE

Adapted for Research on Selective Mutism

SELECTION (max 6 stars)

1) Sampling Method and Representativeness
   a) Truly representative of the average in the target population due to random sampling. ★★
   b) Non-random sampling method but satisfactory representativeness (e.g. clinical referrals). ★
   c) Biased on the part of the participants (e.g. volunteer sampling).
   d) The sample is not representative at all.

2) Sample Size
   a) Large sample size. ★★
   b) Satisfactory sample size. ★
   c) Small sample size.

3) Demographic Distribution
   a) All demographic characteristics are equally distributed. ★★
   b) One demographic characteristic can be considered overrepresented in the sample. ★
   c) The sample is unequally distributed on multiple demographic characteristics.

ASSESSMENT (max 6 stars)

1) Assessment methods of Selective Mutism
   a) Excellent assessment method (e.g. multiple methods including validated structured interviews and clinical evaluations). ★★
   b) Satisfactory or acceptable assessment method (e.g. one validated structured interview or multiple semi-structured interviews). ★
   c) Inadequate assessment method.

2) Informants
   a) Ideally considering independency, clinical experience and number of informants. ★★
   b) Satisfactory despite some limitations in clinical experience, independency or number of informants. ★
   c) Unacceptable (e.g. self-assessment).

3) Procedure
   a) Direct and sufficient assessment procedure (e.g. face-to-face assessment at clinic). ★★
   b) Indirect but satisfactory assessment procedure (e.g. referred assessment or retrospective case evaluation). ★
   c) Unsatisfactory assessment procedure.

Note.

This form is a modification of the Newcastle-Ottawa Scale for assessing the methodological quality of studies in meta-analysis (Wells et al., 2014). Only methods related to sample selection and diagnostic assessment were considered relevant to our research objective. We therefore modified this scale to focus on these methodological aspects only. The original version of the Newcastle-Ottawa Scale can be found on the official website of the Ottawa Hospital Research Institute: www.ohri.ca.

REFERENCES

Alyanak, B., Kılınçaslan, A., Harmancı, H. S., Demirkaya, S. K., Yurtbay, T., & Vehid, H. E. (2013). Parental adjustment, parenting attitudes and emotional and behavioral problems in children with selective mutism. *Journal of Anxiety Disorders, 27*(1), 9-15. doi:10.1016/j.janxdis.2012.10.001

Andersson, C. B., & Thomsen, P. H. (1998). Electively mute children: An analysis of 37 Danish cases. *Nordic Journal of Psychiatry, 52*(3), 231-238. doi:10.1080/08039489850139157

Arie, M., Henkin, Y., Lamy, D., Tetin-Schneider, S., Apter, A., Sadeh, A., & Bar-Haim, Y. (2007). Reduced Auditory Processing Capacity during Vocalization in Children with Selective Mutism. *Biological Psychiatry, 61*(3), 419-421. doi:10.1016/j.biopsych.2006.02.020

Bar-Haim, Y., Henkin, Y., Ari-Even-Roth, D., Tetin-Schneider, S., Hildesheimer, M., & Muchnik, C. (2004). Reduced auditory efferent activity in childhood selective mutism. *Biological Psychiatry, 55*(11), 1061-1068. doi:10.1016/j.biopsych.2004.02.021

Black, B., & Uhde, T. W. (1995). Psychiatric Characteristics of Children with Selective Mutism: A Pilot Study. *Journal of the American Academy of Child and Adolescent Psychiatry, 34*(7), 847-856. doi:10.1097/00004583-199507000-00007

Carbone, D., Schmidt, L. A., Cunningham, C. C., McHolm, A. E., Edison, S., St. Pierre, J., & Boyle, M. H. (2010). Behavioral and Socio-emotional Functioning in Children with Selective Mutism: A Comparison with Anxious and Typically Developing Children Across Multiple Informants. *Journal of Abnormal Child Psychology, 38*(8), 1057-1067. doi:10.1007/s10802-010-9425-y

Chavira, D. A., Shipon-Blum, E., Hitchcock, C., Cohan, S., & Stein, M. B. (2007). Selective Mutism and Social Anxiety Disorder: All in the Family? *Journal of the American Academy of Child and Adolescent Psychiatry, 46*(11), 1464-1472. doi:10.1097/chi.0b013e318149366a

Cholemkery, H., Mojica, L., Rohrmann, S., Gensthaler, A., & Freitag, C. M. (2014). Can Autism Spectrum Disorders and Social Anxiety Disorders be Differentiated by the Social Responsiveness Scale in Children and Adolescents? *Journal of Autism and Developmental Disorders, 44*(5), 1168-1182. doi:10.1007/s10803-013-1979-4

Dow, S. P., Sonies, B. C., Scheib, D., Moss, S. E., & Leonard, H. L. (1995). Practical Guidelines for the Assessment and Treatment of Selective Mutism. *Journal of the American Academy of Child and Adolescent Psychiatry, 34*(7), 836-846. doi:10.1097/00004583-199507000-00006

Dummit, E. S., Klein, R. G., Tancer, N. K., Asche, B., Martin, J., & Fairbanks, J. A. (1997). Systematic Assessment of 50 Children With Selective Mutism. *Journal of the American Academy of Child and Adolescent Psychiatry, 36*(5), 653-660. doi:10.1097/00004583-199705000-00016

Edison, S. C., Evans, M. A., McHolm, A. E., Cunningham, C. E., Nowakowski, M. E., Boyle, M., & Schmidt, L. A. (2011). An Investigation of Control Among Parents of Selectively Mute, Anxious, and Non-Anxious Children. *Child Psychiatry and Human Development, 42*(3), 270-290. doi:10.1007/s10578-010-0214-1

Gensthaler, A., Khalaf, S., Ligges, M., Kaess, M., Freitag, C. M., & Schwenck, C. (2016). Selective mutism and temperament: the silence and behavioral inhibition to the unfamiliar. *European Child and Adolescent Psychiatry, 25*(10), 1113-1120. doi:10.1007/s00787-016-0835-4

Kristensen, H. (2000). Selective Mutism and Comorbidity With Developmental Disorder/Delay, Anxiety Disorder, and Elimination Disorder. *Journal of the American Academy of Child and Adolescent Psychiatry, 39*(2), 249-256. doi:10.1097/00004583-200002000-00026

Lang, C., Nir, Z., Gothelf, A., Domachevsky, S., Ginton, L., Kushnir, J., & Gothelf, D. (2016). The outcome of children with selective mutism following cognitive behavioral intervention: a follow-up study. *European Journal of Pediatrics, 175*(4), 481-487. doi:10.1007/s00431-015-2651-0

Levin-Decanini, T., Connolly, S. D., Simpson, D., Suarez, L., & Jacob, S. (2013). Comparison of Behavioral Profiles for Anxiety-Related Comorbidities including ADHD and Selective Mutism in Children. *Depression and Anxiety, 30*(9), 857-864. doi:10.1002/da.22094

Manassis, K., Tannock, R., Garland, E. J., Minde, K., McInnes, A., & Clark, S. (2007). The Sounds of Silence: Language, Cognition, and Anxiety in Selective Mutism. *Journal of the American Academy of Child and Adolescent Psychiatry, 46*(9), 1187-1195. doi:10.1097/chi.0b013e318076b7ab

Mulligan, C. A., Hale, J. B., & Shipon-Blum, E. (2015). Selective Mutism: Identification of Subtypes and Implications for Treatment. *Journal of Education and Human Development, 4*(1), 79-96. doi:10.15640/jehd.v4n1a9

Oerbeck, B., Stein, M. B., Pripp, A. H., & Kristensen, H. (2015). Selective mutism: follow-up study 1 year after end of treatment. *European Child and Adolescent Psychiatry, 24*(7), 757-766. doi:10.1007/s00787-014-0620-1

Vecchio, J., & Kearney, C. A. (2009). Treating Youths With Selective Mutism With an Alternating Design of Exposure-Based Practice and Contingency Management. *Behavior Therapy, 40*(4), 380-392. doi:10.1016/j.beth.2008.10.005

Vecchio, J. L., & Kearney, C. A. (2005). Selective Mutism in Children: Comparison to Youths With and Without Anxiety Disorders. *Journal of Psychopathology and Behavioral Assessment, 27*(1), 31-37. doi:10.1007/s10862-005-3263-1

Wells, G. A., Shea, B., O'Connell, D., Peterson, J., Welch, V., Losos, M., & Tugwell, P. (2014). The Newcastle-Ottawa Scale (NOS) for assessing the quality of nonrandomized studies in meta-analyses. Retrieved from http://www.ohri.ca/programs/clinical_epidemiology/oxford.asp

Young, B. J., Bunnell, B. E., & Beidel, D. C. (2012). Evaluation of Children With Selective Mutism and Social Phobia: A Comparison of Psychological and Psychophysiological Arousal. *Behavior Modification, 36*(4), 525-544. doi:10.1177/0145445512443980
